# Antibody Labelling of Resilin in Energy Stores for Jumping in Plant Sucking Insects

**DOI:** 10.1371/journal.pone.0028456

**Published:** 2011-12-07

**Authors:** Malcolm Burrows, Jolanta A. Borycz, Stephen R. Shaw, Christopher M. Elvin, Ian A. Meinertzhagen

**Affiliations:** 1 Department of Zoology, University of Cambridge, Cambridge, England, United Kingdom; 2 Department of Psychology and Neuroscience, Dalhousie University, Halifax, Nova Scotia, Canada; 3 Division of Livestock Industries, Commonwealth Scientific and Industrial Research Organisation, St Lucia, Queensland, Australia; University of Arkanas, United States of America

## Abstract

The rubbery protein resilin appears to form an integral part of the energy storage structures that enable many insects to jump by using a catapult mechanism. In plant sucking bugs that jump (Hemiptera, Auchenorrhyncha), the energy generated by the slow contractions of huge thoracic jumping muscles is stored by bending composite bow-shaped parts of the internal thoracic skeleton. Sudden recoil of these bows powers the rapid and simultaneous movements of both hind legs that in turn propel a jump. Until now, identification of resilin at these storage sites has depended exclusively upon characteristics that may not be specific: its fluorescence when illuminated with specific wavelengths of ultraviolet (UV) light and extinction of that fluorescence at low pH. To consolidate identification we have labelled the cuticular structures involved with an antibody raised against a product of the *Drosophila* CG15920 gene. This encodes pro-resilin, the first exon of which was expressed in *E. coli* and used to raise the antibody. We show that in frozen sections from two species, the antibody labels precisely those parts of the metathoracic energy stores that fluoresce under UV illumination. The presence of resilin in these insects is thus now further supported by a molecular criterion that is immunohistochemically specific.

## Introduction

Resilin is found largely in insects and crustaceans but has features in common with other elastic proteins that occur more widely [1]. It consists of coiled peptide chains cross-linked in the mature protein by the peptides dityrosine and trityrosine into a stable, isotropic, three dimensional network [2], [3], [4]. These give it a characteristic fluorescence when illuminated with specific wavelengths of UV light. Pure resilin is a mechanically highly deformable rubber that is elastic even to long range extension and shows almost perfect elastic recovery [5]. For example, the tendon of the pleuro-alar muscle of the dragonfly *Aeshna* can be held at twice its length for a month without experiencing creep only to return rapidly to its original length once the load is removed [5]. Energy loss from resilin during movements, even at 200 Hz, is less than 5% [6] suggesting that it can act as a highly efficient return spring over a wide range of velocities, conserving energy in highly rhythmic movements such as those produced by the flight muscles of insects. In similar fashion, cicadas have resilin in their sound-producing tymbals [7] and some can produce sharply resonant pulses of sound at 13 kHz [8]. Among crustaceans, the flagella of the maxillipeds of crabs and crayfish are moved rhythmically in one direction by a single muscle, while the return stroke is brought about by a resilin spring [9].

Resilin also performs in a different capacity at locations where large amounts of elastic energy storage are required for sudden, one-time release [10]. To power the legendary jumping movements of their hind legs, fleas are suggested to store energy in two pads of resilin in the internal skeleton of the thorax [11], [12], [13]. Click beetles may also use resilin to store some energy for jumping [14] propelled by movements of their thoracic segments [15], [16].

The most completely analyzed examples in this high power category come from study of the jumping prowess of froghoppers and planthoppers (Hemiptera Auchenorrhyncha). These most accomplished jumpers of all insects, depend on the incorporation of resilin into hard cuticle to form a composite material that can store energy [17]. Slow contractions of huge jumping muscles bend these composite energy stores, following which their sudden recoil releases the stored energy in a catapult mechanism that propels these rapid and powerful movements [17], [18]. By itself, the resilin is only able to meet a small percentage of the energy needs, but its rubber-like properties endow the composite material with an ability to resist fracture and return it rapidly to its original shape after the initial energy-storing distortions.

The presence and correct identification of resilin has thus become a key indicator that a structure can be used as an energy store for powering movements. Only two characteristic signatures are currently available for the identification of resilin and both depend upon its fluorescence properties. First, it fluoresces bright blue, with a characteristic wavelength emission under a specific range of excitation with ultraviolet illumination [4], [5]. Second, the intensity of the fluorescence is dependent on the pH of its bathing solution, decreasing when acid and increasing when alkaline [2], [5], [19]. On these criteria alone, identification remains somewhat uncertain, because other biological materials also fluoresce in a similar wavelength range.

Comparisons between the amino acid sequences of tryptic peptides from locust resilin and those predicted from *Drosophila melanogaster* gene products first implicated a *Drosophila* gene present as a single copy, CG15920 [20], [21]. The product of this gene, pro-resilin, has a predicted structure with two long series of amino-acid repeats, A and B, each relatively short and separated by a 68-residue R&R consensus sequence [22] having predicted chitin-binding properties. The consensus sequence coded by CG15920 is the RR-2 form, one of two types found in cuticular proteins, both of which can bind chitin filaments [23]. A second form, RR-1, usually occurs in soft cuticles, however, whereas the RR-2 type apparently occurs preferentially in hard cuticles [24]. The presence of the RR-2 form, in a louse as in *Drosophila* [24], would be in partial agreement with our previous calculations for the other hemipteran species used here, that the pleural arches which power jumps must be formed from stiff cuticle, even if there is no evidence that these are actually sclerotized [17].

To identify resilin protein and its location more closely, we used this fly orthologue provided by the gene CG15920 in *Drosophila melanogaster*. The first exon of this gene has been cloned in *Escherichia coli,* in which it expressed a soluble protein, which when cross-linked formed a resilient, rubbery hydrogel called Rec-1 resilin [25]. Furthermore, an affinity-purified polyclonal antibody raised against this gene product labels locations that are thought to contain resilin in dragonflies [25] and fleas [26], [27]. These authors showed that this anti-Rec1 resilin polyclonal sera is crossreactive with resilin from a distant insect order (Siphonaptera) and predicted that the antibody would represent a valuable resource for future identification of resilin-containing structures within a range of insects. We have therefore sought to determine whether this antibody can be used to identify the location of resilin in the energy storage sites that have been well characterised in particular jumping insects. We show that the labelling of this antibody matches the fluorescence properties of the storage structures thought to contain resilin in two species of jumping bugs (Hemiptera, Auchenorrhyncha). The antibody confirms precisely the earlier identification [17], and provides a third defining characteristic with a specific molecular basis by which resilin can be recognised in future experimental studies in other invertebrates.

## Materials and Methods

Jumping plant bugs, *Philaenus spumarius* (Hemiptera, Auchenorrhyncha, Cercopoidea, family Cercopidae) and *Delphacodes* sp. (Hemiptera, Auchenorrhyncha, Fulgoroidea, family Delphacidae) were collected in and around Halifax, Nova Scotia, Canada in September and October 2010. With the local surface cuticle opened to permit fluid entry, they were fixed for 4 h in 4% formaldehyde, from paraformaldehyde, in 0.1M phosphate buffer (PB), then left overnight in 25% sucrose solution in PB saline. The preparations were next submerged in Tissue-Tek OCT (Andwin Scientific, Addison IL, USA), frozen in liquid nitrogen, and the metathorax cut into cross sections at thicknesses between 10 and 50 µm on a cryostat (Reichert-Jung 2800 Frigocut N). The sections were then rehydrated for 25 min in a 0.25% solution of Triton-X in PB (PBT), permeabilized in 2% PBT for 10 min, then for a further 10 min in 0.25% PBT. They were then blocked in 5% normal goat serum (NGS) for 30 min and incubated overnight at 4°C in a polyclonal anti-rec-1 antibody, at a dilution of 1∶100 in 2.5% NGS. This antibody had been raised in rabbits to a recombinant protein (derived from the first exon of the *Drosophila melanogaster* resilin gene CG15920) expressed in *E. coli* [25]. The next day the sections were brought to 25°C, washed in 0.25% PBT for 30 min, then incubated overnight at 4°C in a 1∶400 dilution of a secondary antibody, goat anti-rabbit conjugated to Cy3 (Jackson ImmunoResearch, West Grove, PA, USA). They were finally washed for 30 min in 0.25% PBT, mounted in a drop of Vectashield (Vector Labs, Burlingame, CA, USA), and viewed with a Zeiss LSM510 confocal microscope using a 10X/0.3 NA or a 25X/0.8 NA (under oil) Plan Neofluar objective lens.

To validate the protein-specificity of a fluorescent label it is essential to check the specificity of the antibody to the antigen [28], for which the following preadsorption controls were therefore performed. The synthetic resilin fraction against which the antibody was raised was diluted to concentrations of 10^−3^, 10^−4^ and 10^−5^ and each was then mixed with the primary antibody solution, at the same concentration of 1∶100 as used for direct labelling. The antigen-antibody mixture was allowed to stand for 24 h in a refrigerator at 4°C and was then centrifuged for 30 min at 20,000 *g*. The supernatant (the putatively preadsorbed serum) was then used in place of the primary antibody in the same immunocytochemical procedure described above, and the sections viewed in the same confocal microscope.

The same sections labelled with the primary and secondary antibodies were also examined and photographed on an Olympus BX51WI compound microscope with a MPlan10X/0.25 NA objective under ultraviolet (UV) or white epi-illumination. Images were captured with a MicroPublisher 5.0 digital camera (QImaging, Marlow, Bucks., UK) as colour (RGB) TIFF files. The UV excitation was provided by an Olympus U-LH100HGAPO, 100 W mercury arc, conditioned by a Semrock DAPI-5060B Brightline series UV filter set (Semrock, Rochester, NY) within a sharp-edged excitation band from 350 nm to 407 nm (1% transmission limits). The resulting blue fluorescence emission was collected in a similarly sharp-edged band at wavelengths from 413 nm to 483 nm through a dichromatic beam splitter and an emission filter. Images captured at the same focal planes under UV and visible light were superimposed in Canvas 12 (ACD Systems of America, Miami, FL, USA). Images of the antibody labelling and those illuminated by UV light from the two microscopes were superimposed in the same way.

## Results

**Figure 1:**
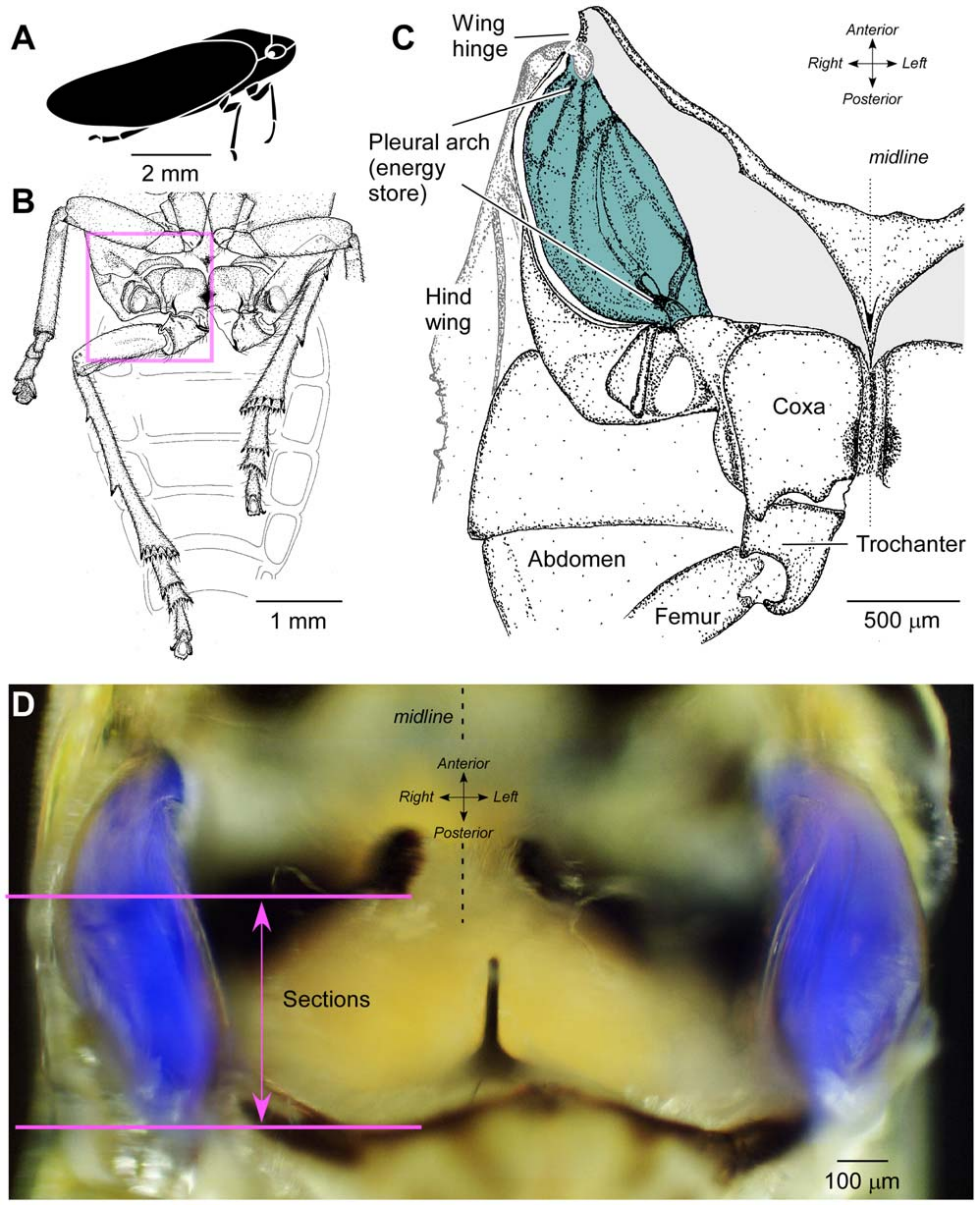
Location of the thoracic energy stores in the pleural arch. A. Cartoon of the froghopper *Philaenus* as viewed from the side. B. Ventral view of the posterior part of the thorax of *Philaenus*. The right hind leg is shown partly depressed and the left hind leg levated with its femoro-tibial joint between the ventral surface of the body and the femur of the left middle leg, held as in preparation for a jump. C. Ventral view of the right half of the metathorax of *Philaenus* (area indicated by box in B) dissected to reveal the massive pleural arch (tinted grey-blue) which with its counterpart on the other side of the body forms the energy store for jumping. D. Photograph of a ventral view of the metathorax of *Delphacodes* in which images illuminated with white and UV light have been superimposed to show the blue fluorescence of the pleural arches. The horizontal lines indicate the region from which the transverse sections shown in subsequent figures were taken.

The energy stores for jumping in *Philaenus* (Fig. 1A) are enlarged internal parts of the skeleton derived from the paired pleural arches (Fig. 1B,C) [17]. The internal anatomy of the energy stores in *Delphacodes* has not been described in detail, but is similar to that of the planthopper *Issus* (Auchenorrhyncha, Fulgoroidea, family Issidae) [29]. A bow-shaped pleural arch links the coxa of each hind leg ventrally to the articulation of the ipsilateral hind wing dorsally (Fig. 1C). When the large trochanteral depressor muscles in the thorax contract slowly in advance of a jump, both pleural arches bend, but the hind legs do not move from their cocked positions. Bending of the pleural arches, which are built of a composite of hard cuticle and what has hitherto been reported as the soft elastic protein resilin, stores energy bilaterally. The sudden release of this energy powers the rapid and simultaneous depression of both hind legs and propels jumps to high take-off velocities [29], [30].

**Figure 2:**
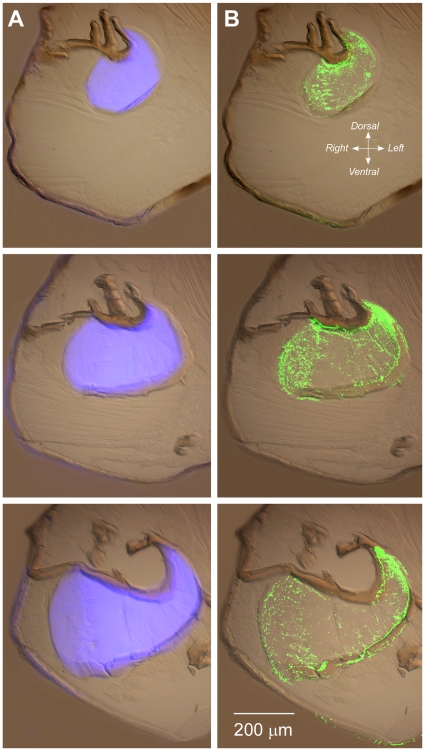
Transverse frozen sections from a series taken through the right half of the thorax of *Philaenus* at the planes indicated in **Figure 1D** and viewed from their anterior surfaces. Dorsal is to the top and ventral to the bottom of each image. A. Superimposed images of the same sections taken with UV and with bright field illumination. Intense blue fluorescence occurs in the pleural arch (energy store) within the thorax. Weaker blue fluorescence is present in the exoskeleton. B. The same sections in which antibody labelling is superimposed on the bright field image. The immuno-signal is restricted to the fluorescent regions with a much weaker signal in parts of the exoskeleton.

In whole animals with the surface cuticle dissected away locally, substantial parts of a pleural arch fluoresced bright blue when illuminated in UV within a specific wavelength range (Fig. 1D). The blue fluorescence began at the articulation with a hind coxa but stopped short of the articulation with a hind wing. This fluorescence has been taken as a key signature identifying resilin. Transverse sections through the metathorax containing a pleural arch also showed this blue fluorescence (Fig. 2A). Within the thoracic cavity the fluorescence was limited to the pleural arches in sharp-edged profiles which changed in shape as the sections passed through different levels. Some blue fluorescence was present in the cuticle of the exoskeleton of the body wall, but this was, by comparison, far less intense.

In sections of the thorax of *Philaenus* treated with the anti-rec-1 antibody, the green signal shown in Fig. 2B from the secondary antibody (converted for clarity from the original red fluorescence of the CY3 fluorophore) always overlapped completely with the region that showed blue fluorescence under UV illumination and this immuno-signal was not present in the remainder of the thoracic cavity. The labelling was patchy and particulate over the surface of the sectioned pleural arch but contrasted strongly with the lack of labelling on other tissues within the thorax. A weaker sprinkling of antibody labelling was also present in some parts of the body wall.

**Figure 3:**
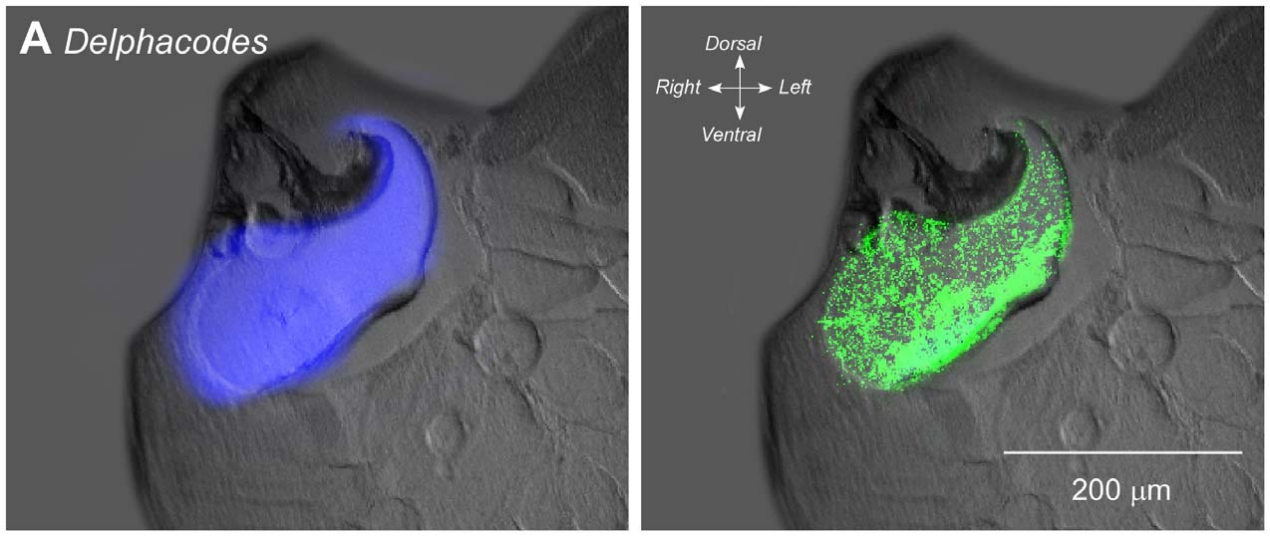
Combined fluorescence and antibody staining of the pleural arches (energy stores) in transverse sections of the thorax of *Delphacodes*. Combined images of bright field and UV fluorescence are shown on the left and of UV fluorescence and antibody labelling on the right.

The same exact covariation of the antibody labelling with the blue fluorescence was also seen in *Delphacodes* (Fig. 3). As with *Philaenus*, blue fluorescence under UV illumination again was restricted to the pleural arches, and the antibody labelling co-localized accurately to these structures. Weaker antibody labelling was also present in parts of the body wall.

**Figure 4:**
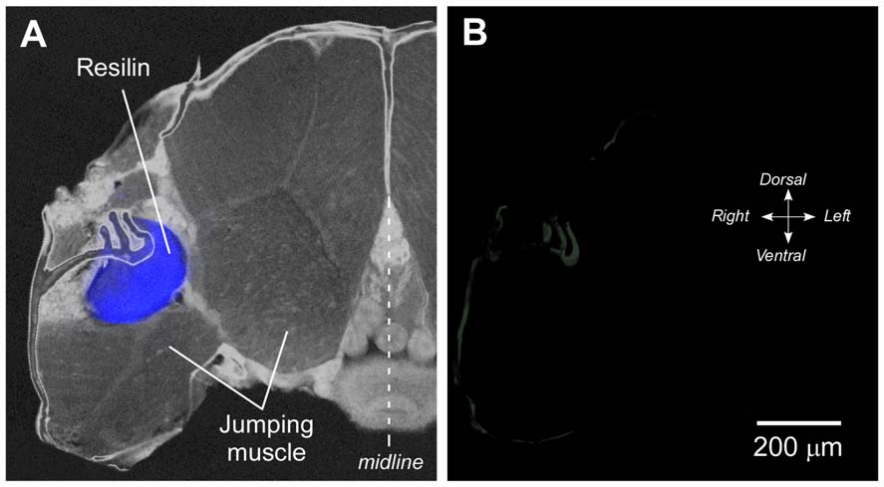
Preadsorption controls to show the specificity of the antibody. A. Transverse section of the thorax of *Philaenus* illuminated with bright field and UV light. Blue fluorescence is restricted to the pleural arch (energy store). The large muscles that power depression of the hind legs in jumping occupy most of the volume of the thorax. B. Incubation in the antibody after preadsorption with the antigen now fails to label the pleural arch. Only some weak immunolabelling is still present in the exoskeleton.

To test the specificity of the antibody, preadsorption controls were performed (see Methods section). No labelling occurred when the primary antibody was replaced with the preadsorbed serum at all three dilutions (10^−3^ to 10^−5^) of the antigen used (Fig. 4A,B). Some weak labelling remained in the cuticle of the body wall. Thus, our finding of specific labelling by an antibody raised against an integral part of the resilin gene product at the exact sites of the blue fluorescence, confirms by means of an exacting test at the molecular-level that the blue fluorescence previously ascribed to resilin does indeed derive from resilin protein.

## Discussion

The parts of the energy stores for jumping that fluoresce bright blue when illuminated with specific wavelengths of UV light were also labelled specifically by an antibody raised against the product of an integral part of the only resilin gene in *Drosophila*, Rec-1 resilin. No other structures within the body were labelled although some weaker immunolabelling was present in the body wall. Preadsorbing the antibody with the antigen abolished all labelling of the energy stores but some residual labelling remained in the body wall. This residuum could be the result of autofluorescence or more likely indicates a property of the secondary antibody used. The clear and precise matching of the antibody labelling with the blue fluorescence indicates that both techniques reveal the same target, the elastic protein resilin.

Antibody labelling of the energy stores was typically patchy. While the blue resilin fluorescence seemed to emanate continuously from within the pleural arch as determined by through-focussing, in contrast, antibody labelling appeared to be more patchy. This distribution was, however, not expressed systematically on a microscale, and its punctate appearance varied between adjacent sections from each preparation. This patchiness is attributed to a lack of penetration of the large antibody molecules into the thick, hard cuticle of the pleural arch, such that only the surface of the sections were labelled: we previously found that penetration even of small aqueous-phase hydrogen ions into unfixed pleural arches *in vivo* was slow and incomplete [17]. Insect cuticle varies widely in its state of hydration at different locations [31], so poor penetration of a weakly hydrated cuticle would not be unexpected. Additionally, the density of antigenic binding sites to be expected at the surface of a section is unclear, for an antibody raised against only a small part of the large amorphous native protein. On a coarser viewing scale though, much of the labelling seemed to be located towards the edges of the pleural arches (Fig. 2B). Although this remains to be confirmed in quantitative detail, these peripheral locations are those that should experience the greatest stress during bending and release of the arches during jumping. These might therefore be the best places to concentrate a larger amount of resilin cross linked to cuticle, given its suggested role in anti-shatter and quick-recovery functions in enabling the huge jumps made by these insects [17].

### What is the antibody labelling?

The antibody was raised against the Rec-l resilin product of the *Drosophila melanogaster* gene CG15920, of which only the first exon was expressed in *E. coli* [25]. One of two repeats that confers elasticity on the protein, the ‘A-repeat’ sequences in this region consist of multiple short motifs invariant except at the last 3 positions (xxx), GGRPSDSYGAPGxxx, while the number of these repeats varies from 13 to 18 in twelve different species of *Drosophila* [24]. Given its cross reactivity demonstrated in different species, both ancient, Odonata [25], and two more recently evolved, Siphonaptera [26], [27], and now in this report, Hemiptera, the antibody is predicted to recognise those repeats that have been conserved in different insects. Testifying further to the prospect of broad-spectrum recognition by the antibody, the odonatan example comes from the resilin subgroup which has a return spring function and does not co-extract with cuticular binding proteins, while the two other examples come from the other resilin subgroup that is associated with cuticular distortion, for instance during jumping, and was co-extracted with cuticle in one example examined [24]. A search of the recently released genome of the mosquito *Anopheles gambiae* for the YGAP part of the A-repeat sequence resulted in discovery of a single copy of another pro-resilin gene. A synthetic construct based on the consensus repeat unit of that gene has been developed, and the resulting recombinant protein (AN16) expressed in *E. coli* [32]. This second protein also can be cross-linked via dityrosine bonds to produce a hydrogel with similar mechanical properties to those of the Rec-l protein. Natural resilin in both fly species has a highly amorphous structure as seen in TEM images (there is no evidence of structure) [33], and NMR and circular dichroism studies of solutions of synthetic resilin produced by the expression of a resilin gene concur in implying that it is largely unstructured [34]. A synthetic recombinant resilin AN16 (based on a putative resilin gene found in the genome of *Anopheles gambiae*) shows little evidence of secondary structure [34]. Computer modelling analysis suggests that the chitin-binding (RR domain) region of resilin proteins display some ordered (beta-strand) structure that may persist in the presence of chitin fibrils [35].

The blue fluorescence attributed to resilin when illuminated with specific UV wavelengths has been found in a much wider range of insects and crustaceans than the insects examined so far with the anti-rec-1 antibody. If this too reveals resilin, as seems likely, must this imply that resilin possesses the same properties wherever it occurs in arthropods?

### Resilin in different insects

Pro-resilin gene orthologues have been identified in 12 *Drosophila* species and these show sequence similarities to related genes from other insect groups [24]. The group of pro-resilin like proteins has been divided into two subgroups, that display one or both of two defining features, long-range deformability combined with elastic recovery associated with multiple proline- and glycine-rich repeats, and a 68 residue chitin-binding zone, the R&R consensus region. Reflecting the range of their possible functions, candidates - including one from the mosquito *A. gambiae* - lack the latter region and thus may not be cuticular proteins at all [24].

The various roles that resilin plays as a cuticular protein in many insects indicate that at least the requirements for stiffness associated with this protein must differ. Resilin is present in tendons, at the articulations of hairs with their sockets, in the membranes of limb joints, and in energy storage devices for jumping. The varying mechanical requirements for the functions that resilin must subserve at these different locations could be met in a number of ways.

First, the fractional amount of resilin that is present clearly varies enormously. For example, in the pleural arches of froghoppers the quotient of the volume of resilin per body mass is 2.3 mm^3^/g, more than 10 times greater than that for the pads associated with the hind legs of fleas [11], and much greater still when compared with the amounts present in a cockroach leg [19] or a cicada tymbal [36].

Second, the stiffness of the resilin should be changed by altering the density of its dityrosine cross links. There are no direct measurements of the stiffness of resilin in different locations in different insect species to indicate whether this option has been exploited, but the percentage of tyrosine in known resilin-like proteins varies from 0.4 to 12.3 percent [24], suggesting some potential.

Third, resilin may be incorporated into composites or metamaterials, through the chitin binding domains present in some resilin molecules [37], now identified as the R&R consensus sequence [22], [24]. The use of composites appears to be the key to energy storage in the structures analysed here [17]. The ratio of resilin to cuticle at different sites could also vary adaptively, but measurements exploring this possibility are so far lacking. The observation here of increased antibody binding at the periphery of the pleural arches (Fig. 2B) may indicate that a higher concentration of resilin-cuticle forms a more flexible reinforcing shell.

So far it seems that resilin is used in two distinct ways by insects. In the first, it acts as an energy-conserving buffer for rhythmically active, fast mechanical movements, such as those of the wings during flight, or the tymbals used in cicadas to generate sound [7]. In these examples, its resilience allows it to return nearly all of the energy stored for the next cycle of movement [5]. The second role, as a device for storing huge amounts of energy, is in providing a flexible material that combines with the stiffer, power storing chitinous cuticle as a composite structure. The resultant properties of the composite then become very different from the properties of the individual constituents. Stiff cuticle can store much energy when bent by only a small amount, while the soft, cross-linked resilin prevents it from fracturing. The resilience of resilin then restores body shape after a jump, allowing further jumps to be made with minimal delay.

In all these examples the mechanics of the movements are strongly influenced by structural components. In turn the latter exert profound constraints on the motor patterns that need to be generated by the nervous system and on the performance of the muscles. The execution of a successful movement depends on the interactions between all elements, with the mechanical construction often providing solutions to problems that would be complex for the nervous and muscular systems to solve alone. It is thus critical to be able to identify the presence of key materials such as resilin within body structures and to consider the role these may play in the execution of a particular movement. Hitherto, the identification of resilin has been rather loosely based. An antibody that recognises resilin in such a wide range of species thus provides an important diagnostic tool, one that allows resilin to be distinguished from other molecules that may exhibit similar fluorescent characteristics, and is in turn key to the clearer interpretations of how animals are enabled to make movements.
